# Looking outside the box: a comparative cross-kingdom view on the cell biology of the three major lineages of eukaryotic multicellular life

**DOI:** 10.1007/s00018-023-04843-3

**Published:** 2023-07-07

**Authors:** Ralph Panstruga, Wolfram Antonin, Alexander Lichius

**Affiliations:** 1 Unit of Plant Molecular Cell Biology, Institute for Biology I, RWTH Aachen University, Worringerweg 1, 52056 Aachen, Germany; 2 Institute of Biochemistry and Molecular Cell Biology, Medical School, RWTH Aachen University, 52074 Aachen, Germany; 3 inncellys GmbH, Dorfstrasse 20/3, 6082 Patsch, Austria; 4 Department of Microbiology, University of Innsbruck, Technikerstrasse 25, 6020 Innsbruck, Austria

**Keywords:** Cell biology, (Sub-)cellular structures, Cellular functions, Cross-kingdom, Eukaryotic multicellular life, Animals, Fungi, Plants

## Abstract

Many cell biological facts that can be found in dedicated scientific textbooks are based on findings originally made in humans and/or other mammals, including respective tissue culture systems. They are often presented as if they were universally valid, neglecting that many aspects differ—in part considerably—between the three major kingdoms of multicellular eukaryotic life, comprising animals, plants and fungi. Here, we provide a comparative cross-kingdom view on the basic cell biology across these lineages, highlighting in particular essential differences in cellular structures and processes between phyla. We focus on key dissimilarities in cellular organization, e.g. regarding cell size and shape, the composition of the extracellular matrix, the types of cell–cell junctions, the presence of specific membrane-bound organelles and the organization of the cytoskeleton. We further highlight essential disparities in important cellular processes such as signal transduction, intracellular transport, cell cycle regulation, apoptosis and cytokinesis. Our comprehensive cross-kingdom comparison emphasizes overlaps but also marked differences between the major lineages of the three kingdoms and, thus, adds to a more holistic view of multicellular eukaryotic cell biology.

## Introduction

**Fig. 1:**
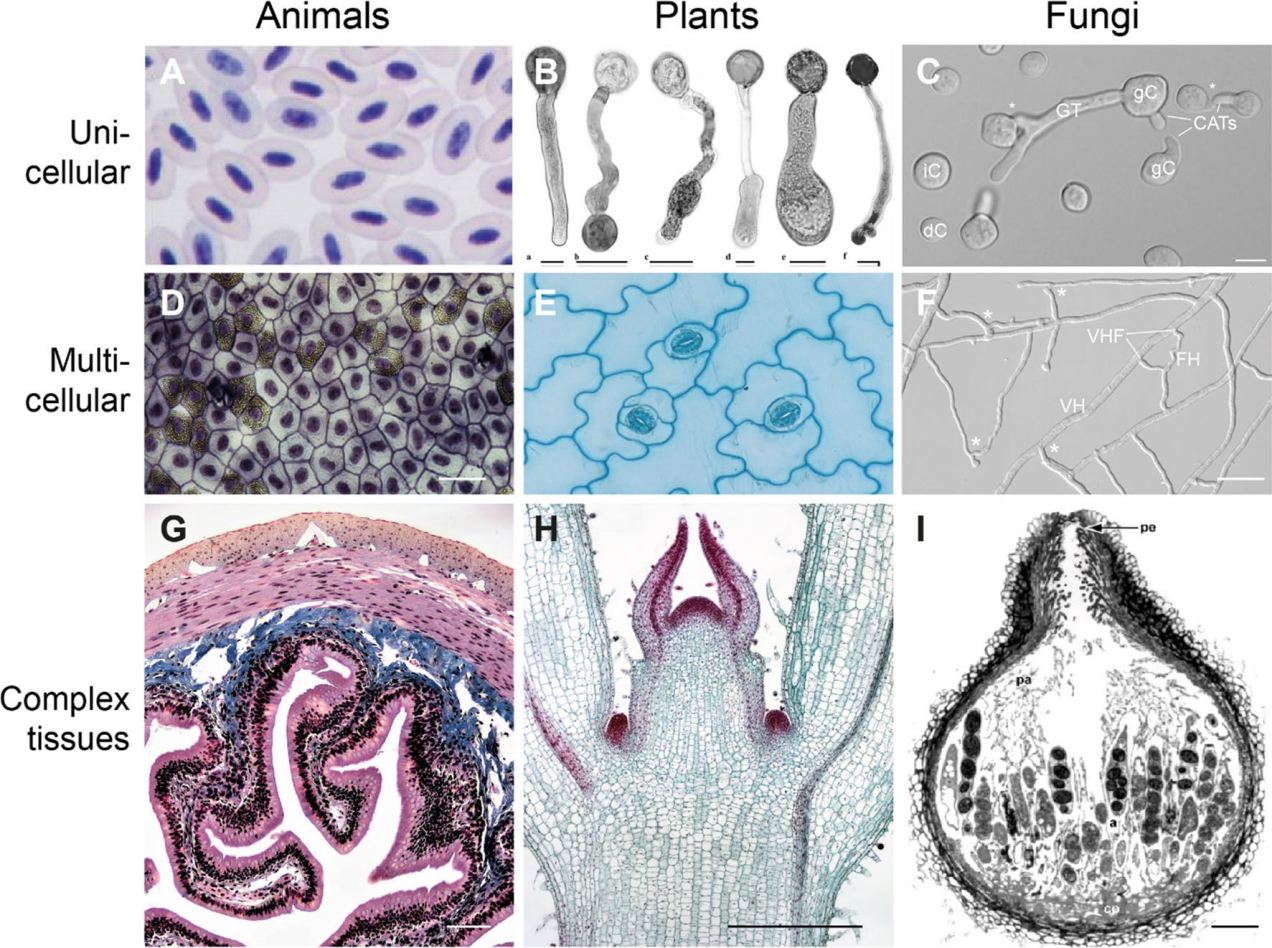
The connection of individual cells to multicellular networks is an essential prerequisite for the development of multifunctional tissues in animals, plants and fungi alike. **A** Giemsa–Wright combination staining of avian red blood cells (courtesy of Jennifer Owen, Michigan State University, MI, USA). **B** Pollen tube photomicrographs of different angiosperm species (reproduced with permission from [33]). Scale bars, 20 µm. **C** Unicellular, dormant fungal spores (conidia, dC) - here shown from *Neurospora crassa - *are the most common starting point of a fungal colony. Isotropic expansion (iC) precedes germination (gC). Conidal germlings differentiate germ tubes (GT) which extend and explore the substrate, as well as conidial anastomosis tubes (CATs) which establish cell–cell fusion connections (*). GT extension and CAT-mediated cell fusion establish an interconnected germling network in which original cells lose their individuality. Hence, the network is regarded as having a supracellular state. Adapted with permission from Ref. [121]. Scale bar, 10 μm. **D** Simple squamous epithelium from a frog (courtesy of Fayette Reynolds, Berkshire Community College, MI, USA). Scale bar, 200 µm. **E** Surface view of lower epidermis of leaf of *Kalanchoe* (courtesy of John Adds, Science and Plants for Schools; https://www.saps.org.uk/). **F** With increasing length, GTs - here shown from *Neurospora crassa - *mature into vegetative hyphae (VH), which branch and differentiate fusion hyphae (FH) to establish vegetative hyphal fusion (VHF) connections (*) that expand the interconnected mycelium network further. Adapted with permission from Ref. [160]. Scale bar, 100 μm. **G** Simple columnar epithelium of a mammalian gut (courtesy of Fayette Reynolds, Berkshire Community College, MI, USA). Scale bar, 500 µm. **H** Staining of a shoot apex of *Coleus blumei* (alias *Solenostemon scutellarioides*) (courtesy of John Hardy, University of Wisconsin—Stevens Point, WI, USA). The red-stained dome-shaped tip of the cone in the center of the micrograph is the apical meristem. Scale bar, 500 µm. **I** The interconnected vegetative mycelium forms the basis for further differentiation. Starvation conditions, for instance, trigger sexual development of female fruiting bodies (perithecia) - here shown from *Sordaria macrospora* - in which highly durable sexual spores (ascospores, AS) develop that can outlast unfavorable conditions. Perithecia comprise different cell and tissue types that form under the participation of cell fusion processes and thus represent some of the most complex multicellular structures that fungi are able to create. (a, asci; cp, centrum pseudoparenchyma cells; pa, paraphyses; pe, periphyses). Modified with permission from Ref. [122]. Scale bar, 25 µm

Cells are the universal building blocks of all organisms—from simpler organized single-celled pro- and eukaryotes to highly complex multicellular species. Eukaryotic cells exhibit extensive compartmentalization: they are substructured by the presence of multiple intracellular organelles, which form dedicated membrane-enclosed reaction chambers for particular biochemical and cell biological processes. Taxonomically, the superkingdom (empire) of eukaryotic life can be subdivided into several kingdoms. According to the traditional view, these include the unicellular protists (Protozoa), Chromista, plants ((Viridi-)Plantae), fungi and multicellular animals (Animalia, metazoa) [176], although recently revised taxonomies draw a more complex picture of the diversity of eukaryotic life, predominantly due to the addition of numerous new “kingdom-level” lineages of heterotrophic protists [2, 26, 100]. Of the traditional kingdoms, according to the number of extant catalogued and predicted species, animals, plants and fungi can be considered as the major kingdoms of multicellular life [136], though multicellular eukaryotes also occur within the Chromista (e.g. marine brown algae and oomycetes). While a majority of cellular features and functions are shared between these three prominent eukaryotic kingdoms, important details also differ. Nevertheless, cell biology textbooks often present facts in a human/animal-focused manner. This apparent bias on the one hand echoes the unequally distributed extent of research activities devoted to the three kingdoms. On the other hand, it probably also reflects the generally homocentric worldview. Accordingly, textbooks typically provide a skewed knowledge of cell biology, which often persists throughout a scientist´s career. We thus see a need to provide a more balanced and holistic view on eukaryotic cell biology. We focus our cell biological comparison presented here on the three major eukaryotic kingdoms of (multicellular) life, i.e. animals (with a focus on vertebrates/humans), (land) plants and (filamentous) fungi, i.e. the multicellular eukaryotic lineages that display the greatest catalogued species richness [151]. While animals and fungi as well as several types of protists can be grouped taxonomically as Opisthokonta (i.e. organisms in which flagellate cells have a single posterior flagellum), plants reside in a different branch in the tree of life and, thus, are more distantly related to both animals and fungi, which likely share a common ancestor. We deliberately exclude unicellular eukaryotes from our comparative analysis as protists exhibit an enormous diversity regarding cellular and molecular characters, largely precluding the elaboration of common principles. In animals, fungi and plants, cells are connected to multicellular networks for the development of multifunctional tissues (Fig. 1). They are further well-studied at the cell biological level and together comprise the majority of known eukaryotic species worldwide [136]. We highlight characteristics that discriminate humans/animals, plants and fungi at the cell biological and molecular level. Given the number of aspects we touch upon, not all of these can be discussed in full depth. We thus refer to the cited literature for further details. We also like to stress that details may differ for specific subgroups of the considered organisms, in particular in early-diverged lineages, and that often exceptions from the general exist, sometimes even within a taxonomic lineage or within a given organism.

## Cellular structures

### Cell size and shape

It is often stated that plant cells are large (10–100 µm) and rectangular while animal cells are small (10–20 µm) and roundish. While this might be true for some cell types, there exist many exceptions in both kingdoms of life. For example, in humans both cell size and shape vary considerably, with the human neutrophils (10 µm) and oocytes (100 µm) being representatives of particularly small and large cells, respectively. Neurons, epithelial cells and muscle cells, for instance, deviate markedly in shape from the archetype of a round animal cell [74]. Likewise, cell size and shape differ in plants. While some plant cell types are indeed rectangular/cylindrical (e.g. many root cell types), others might be rather roundish (e.g. leaf mesophyll cells), jigsaw puzzle-shaped (e.g. epidermal pavement cells) or elongated (e.g. fiber cells). Cell size in plants is also strongly dependent on endoreplication, a process that is more common in plants than in animals [190]. While cells of unicellular yeast-like fungi are typically small (2–10 µm and have an approximately oval shape—with the exception of dimorphic species that can switch between budding and (pseudo-)hyphal growth [23, 182]—cells of generally multicellular filamentous fungi are morphologically much more diverse. In the model ascomycete *Neurospora crassa*, for instance, 28 different cell types have been described [17]. Sexual and asexual spores (conidia) exist in many species-specific shapes and sizes (ranging from 2 to 25 µm in length) adapted to their living and propagation environment [30]. In their vegetative state, filamentous fungi grow as tube-like elongated cells, called hyphae, with an average diameter of 10–15 µm. If not restricted by external factors, such as space, water or nutrient limitation, hyphae can extend indefinitely and have created the humongous fungus, a honey mushroom (*Armillaria ostoyae*) covering 9.6 km^2^ in the Malheur National Forest in Oregon, USA [227]. Until very recently, this fungus held the record as the largest living organism on the planet, however, was now surpassed by Poseidon’s ribbon weed sea grass (*Posidonia australis*) inhabiting an area of 200 km^2^ in Shark Bay, Australia [49]. The definition of a cellular unit is not straightforward in filamentous fungi as both spores and hyphae can be compartmentalized by cross walls, so-called septa (see Sect. “Cell–cell junctions”).

Hyphae of lower fungi (like the order of the Mucorales from the Zygomycota) are non-septate. Consequently, their mycelium can be considered to be unicellular [181]. Hyphae of higher filamentous fungi (like those belonging to the Ascomycota and Basidiomycota) are compartmentalized by septa with central pore openings that allow free cytoplasmic streaming throughout the interconnected cells (syncytium). Therefore, their mycelium can be considered unicellular as well. Nevertheless, closure of septal pores (see Sect. “Organelles”) can physically isolate hyphal compartments and allow the fungus to switch from the unicellular state to a true multicellular organization [18]. Switching between unicellular and multicellular organization is thus a common part of the development of multicellular fungi and their lifestyles and helps these organisms to either adapt to or escape from unfavorable living conditions. Multinucleated cells are also frequent in animals [130]. They can be formed by cell–cell fusion and are then referred to as syncytia. A classic example of a syncytium is the vertebrate skeletal muscle, where muscle fibers form by fusion of thousands of individual muscle cells. Alternatively, multinucleated cells may form by nuclear divisions without cytokinesis, referred to as coenocytes. Many insect embryos such as in *Drosophila* show such incomplete cell divisions during early development, leading to multinucleated blastoderms. These blastoderms are typically later during a process called cellularization cleaved into individual cells. However, coenocytes may also persist as large cells during development [152]. In a tissue context, neighboring plant cells are typically interconnected via cytoplasm-filled channels in their cell walls (plasmodesmata, see Sect. “Cell–cell junctions”). This syncytium-like cytoplasmic space is referred to as the plant symplast [53].

### Cellular protrusions

Due to the lack of a rigid cell wall (see Sect. “Extracellular matrix”), animal cells can have different types of rather plastic cellular protrusions. Common examples are microvilli and pseudopods. Microvilli are finger-shaped plasma membrane projections that primarily serve the purpose of cell surface enlargement. They are mostly found on the apical side of epithelial cells, e.g. in the case of the gastrointestinal tract, where they are involved in nutrient absorption [37]. Pseudopods are temporary actin-rich extensions of the cytoplasm that are typically used for the purpose of cell movement [212]. Depending on their appearance, pseudopods can be further classified in different subtypes such as lamellipodia and filopodia. A distinct form of membrane protrusions called blebbing arises when the plasma membrane locally detaches from the underlying actin cortex [150]. This allows the cytoplasmic pressure to push the membrane outwards and can contribute to cell movement. Cilia constitute permanent, thin, microtubule-based extensions in animal cells [184]. As motile cilia (see Sect. “Motile cilia and flagella”) of epithelial cells they have important functions, e.g. in mucus clearance or moving the egg in the fallopian tube. As immotile or primary cilia, they have a crucial role in signaling and are often described as the cellular antennae.

Plant cells, which are surrounded by stiff cell walls (see Sect. “Extracellular matrix”), rarely show cell protrusions. A notable exception are plant root hairs, which are tubular extensions of root epidermal cells. Similar to the microvilli of animal cells, they greatly increase the cell surface area, promoting water and mineral uptake via the plant root [153]. In addition, actin-rich protrusions form the basis for the lobes in the leaf epidermal cells of some plant species, which may reduce mechanical stress [183] (Fig. 1E). However, in contrast to the short-lived, dynamic and flexible pseudopods of animal cells, root hairs and epidermal cell lobes are static and, once formed, remain essentially invariant.

Filamentous fungi grow by polarized tip growth (see Sect. “Tip growth”). Secretory vesicles deliver new building material for plasma membrane and cell wall extension to drive hyphal tip protrusion [165, 168]. Apical exocytosis is orchestrated with subapical endocytosis to reuse non-incorporated building material, and allows for very high tip extension rates. Fungal spores and mature hyphae are able to adapt their growth morphology to changing environmental conditions very quickly. For example, vegetative spores generate germ tubes and conidial anastomosis tubes [170], and mature hyphae create new side branches and establish hyphal fusion connections [85] (Fig. 1C and 1F). Each polarized growing tip is capable of rapidly redirecting its growth direction in response to chemotropic cues [24, 98]. Once formed, the surrounding cell wall generally rigidifies these structures. Nevertheless, a variety of cell wall remodeling enzymes (see Sect. “Extracellular matrix”) facilitates their reshaping to adapt to alternative developmental pathways, for example, after injury [80].

### Motile cilia and flagella

Motile cilia are miniature, whip-like cellular projections of stationary cells whose beating generates a directional fluid flow. In humans, they are found in the lungs, respiratory tract, fallopian tube and middle ear, but also in cells of the nervous system such as the spinal cord and the brain ventricular system [164]. By contrast, flagella are hair-like structures that primarily serve a role in locomotion. In humans, the only flagellate cell type are the male sperm cells. At the ultrastructural level, both motile cilia and flagella share a common composition: They are built on a scaffold of doublet microtubules and powered by dynein motors (see Sect. “Motor proteins”). The capacity to produce cilia and flagella is maintained in early-diverged plant lineages but has been lost in seed plants in the course of evolution [86]. Flagellated sperm cells are still present in some basal plant lineages such as bryophytes, ferns and gymnosperms. In almost all fungi, cilia have been lost throughout evolution [157]. As an exception in the fungal kingdom, zoospores of the Chytridiomycota are powered by flagella that due to their diverse ultrastructure are used for phylogenetic classification [92]. A recent study highlights the strong correlation between the occurrence of the p25-alpha domain of tubulin polymerization promoting protein-like proteins in eukaryotic cilia/flagella of animals and fungi [147]. In animals, a second type of cilia, immobile or primary cilia, are found as antenna-like protrusions on many excitatory and non-excitatory cells [192]. Although they are, as motile cilia, microtubule-based structures, they act in chemosensation, signal transduction like hedgehog signaling, and probably cell growth control.

### Motile cells

Animals have circulatory systems that host different types of motile cells. In the case of vertebrates, prominent examples are blood cells, which can be further subdivided into oxygen-transporting “red” blood cells (erythrocytes) and “white” blood cells (leukocytes), which engage in different tasks in immunity. Cell motility is mostly passive and based on the blood stream. However, some leukocyte types can leave the blood stream and actively enter into tissues (“extravasation”), especially when attracted to a site of wounding or infection. Single cell migration is also important for developmental processes in metazoans, e.g. during development of the nervous system, but also to maintain tissue homeostasis, while aberrant cell migration is found in various pathologies including cancer cell migration during epithelial-mesenchymal transition [161]. Additionally, collective cell migration is the prevalent mode of migration during development, wound healing, and tissue regeneration [57], with gastrulation arguably representing the most impressive example. Unlike animals, neither plants nor fungi have circulatory systems and their cells also do not show collective migration of cohesive cell groups. Accordingly, these organisms lack motile cells that operate within the organism. Flagellate sperm cells of basal plant and fungal lineages that move through the environment towards mating partners have already been mentioned (see Sect. “Motile cilia and flagella”).

### Contractile cells

Contractile cells are common in animals. They have the capacity to exploit the interaction of cytoskeletal actin filaments and myosin motors (see Sect. “Motor proteins”) for alternating cycles of cell shortening and expansion. Contractile cells can be found in various animal tissues, including the skeletal muscles (striated muscle cells), the heart (heart muscle cells, cardiomyocytes), and the gastrointestinal tract (smooth muscle cells). Also non-muscle cells have contractile capacity due to the presence of actomyosin bundles (stress fibers) that are dispersed in the cell. These structures form to withstand and respond to mechanical stresses [6], constrict cells to divide during cytokinesis [34], and participate in coordinated cell deformations during morphogenesis [141]. Unlike animal cells, plant and fungal cells are non-contractile. The rather rigid cell walls (see Sect. “Extracellular matrix”) that enclose the cells of these organisms prevent any instantaneous cell deformation of recurring contractions and expansions. Nevertheless, very fast constricting cellular structures do exist in fungi. Nematophagous fungi, for instance, produce constricting trapping rings to capture, kill and digest nematodes as food source [198]. Nevertheless, the mechanism of ring closure is based on volume expansion rather than the constriction of individual cells.

### Stem cells and totipotency

Multicellular organisms’ evolution is commonly thought to have been accompanied by the evolution of a stem cell system. Stem cells are self-renewing cells that divide symmetrically and/or asymmetrically either to self-renew or to differentiate into different types of progeny cells. They are usually classified as multipotent if they can develop to multiple cells within a lineage, pluripotent if they can differentiate into all cell types in an adult, and totipotent if they can give rise to an entire organism, i.e. differentiate into any embryonic cell as well as, where relevant, any extraembryonic cell. True totipotency, or even pluripotency, is rare in animals. It is widespread in animal sponge cells [140], but is restricted to neoblasts in planaria [173] and has become increasingly exceptional in higher animals. In mammals, it is restricted from the zygote to early blastomere development, possibly only found for the fertilized oocyte [21]. In contrast, plant cells retain totipotency and developmental plasticity also in their differentiated state. They have the ability to dedifferentiate, proliferate and subsequently regenerate into mature plants under suitable culture conditions in a process called somatic embryogenesis [199]. Apart from that, plants harbor pluripotent stem cells in specialized tissues termed meristems, which are the sites of repeated cell division, giving rise to plant growth [144] (Fig. 1H). Contrary to the rarity of totipotent cells in animals but similar to plants, almost every cell formed by a fungus can function as a “stem cell” [135]. The basic undifferentiated, totipotent cellular element is the compartmentalized vegetative hypha at the colony periphery (the leader hypha) [17]. Its main function is to explore the surroundings and extend the colony on a suitable substrate as quickly as possible. Vegetative hyphae can rapidly differentiate other morphologies, such as aerial hyphae and conidiophores, respectively, to escape unfavorable conditions through the production of conidiospores. Certain other cell types are comprised of differentiated hyphae with dedicated functions (e.g., fusion hyphae, ascogonia, trichogynes, ascogenous hyphae, asci, paraphyses, and periphyses), many of which involved in the formation of sexual reproductive structures. At the other extreme are highly differentiated non-hyphal cells such as ascospores, microconidia, and the different wall cells of protoperithecia and perithecia (see Fig. 1I). Nevertheless, many of the named cell types and hyphal elements are—despite their functional specialization—capable of starting a new fungal colony after being physically separated from the mycelium, provided at least one cellular unit remains intact.

### Extracellular matrix

**Fig. 2:**
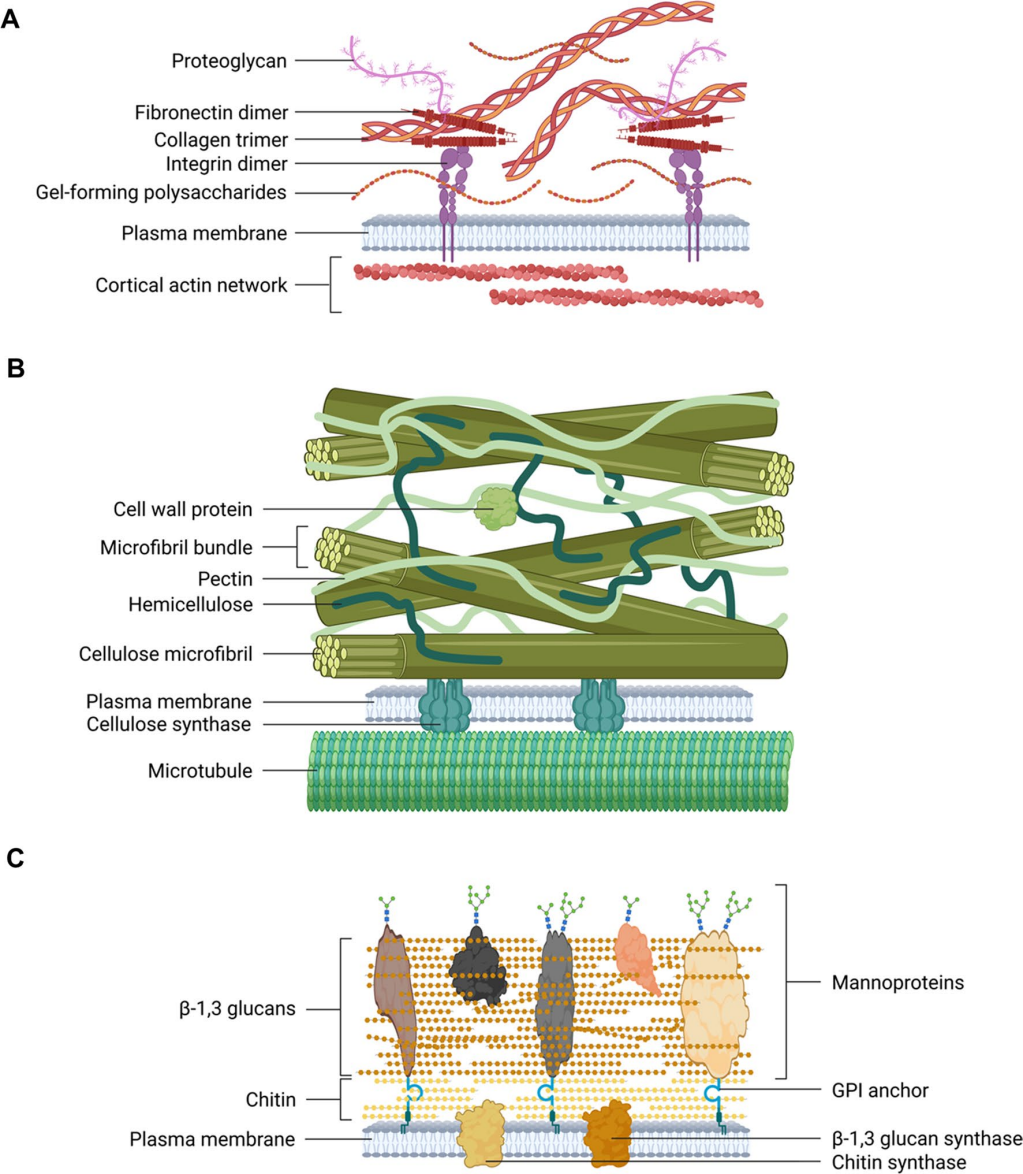
Schematic representations of the extracellular matrix and cell walls of animal, plant, and fungal cells. **A** The animal extracellular matrix is less densely packed than the plant and fungal cell walls and composed of trimeric collagen helices and proteoglycans. These constituents are linked to fibronectin dimers, which are in turn associated with plasma membrane-localized integrin dimers. Gel-forming polysaccharides fill the gaps between collagen and proteoglycans. **B** Primary plant cell wall composition. Primary plant cell walls are composed of densely packed cellulose fibrils (organized in bundles), hemicellulose and pectin. Cellulose molecules are synthesized by plasma membrane-localized cellulose synthase rosette complexes that move along cytoplasmic microtubule tracks. Few cell wall proteins are present in the mainly carbohydrate polymer-based meshwork. Secondary cell walls contain in addition lignin (not shown). **C** Fungal cell walls are composed of a densely packed chitin layer, typically localized below a thicker, densely packed glucan layer, primarily composed of β-1,3 glucans. Chitin- and β-1,3 glucan synthesizing enzymes reside in the plasma membrane. Mannose-rich glycoproteins (mannoproteins), which are in part attached to the outer leaflet of the plasma membrane by glycosylphosphatidylinositol (GPI) membrane anchors, are embedded into the glucan layer. The *N*-acetyl-glucosamine building blocks of chitin enable more inter-chain hydrogen bonds than the glucose subunits of cellulose in the plant cell wall, yielding an overall higher rigidity of the fungal cell wall. Note that the three schemes represent simplified prototypical arrangements. Figure created with BioRender.com

The extracellular matrix (ECM) is a three-dimensional network of macromolecules that surrounds cells to provide structural support and to protect them. The ECM of animal cells is largely composed of proteoglycans (heavily glycosylated glycoproteins), polysaccharides (e.g. hyaluronic acid or chondritin) and proteins (e.g. collagen or laminin) and is typically soft and flexible (Fig. 2A), but in some instances can also be hard such as in the case of endo- and exoskeletons. While several animal cell types are surrounded by/embedded in extracellular matrix, others (such as the mobile blood cells; Fig. 1A) lack an extensive ECM contact. In plant cells, the cell wall can be viewed as a rigid type of ECM that is primarily composed of various types of polysaccharides (cellulose, hemicellulose, pectin) with only a small proportion of proteins (Fig. 2B). The plant cell wall is the prerequisite for the establishment of the osmotically conditioned turgor pressure and for the phenomenon of plasmolysis (the retraction of the protoplast from the cell wall in a hypertonic solution). While the primary walls of plant cells, despite their general rigidity, retain some plasticity and allow for the turgor pressure-driven expansion of plant cells, lignified secondary cell walls of supporting cell tissues such as sclerenchyma and water-conducting xylem elements are much more rigid [11]. Similar to plant cells, fungal cells are likewise surrounded by a shape-determining cell wall composed of different types of β-glucans, chitin and glycoproteins (Fig. 2C) [177]. Notably, due to an extensive portfolio of cell wall-modifying enzymes, fungi can remodel their own cell walls—and that of other fungi and plants—in order to adapt their morphology to changes in the environment and to interact with host organisms such as symbionts or parasites [64, 73, 112]. Similarly, plants possess a broad collection of proteins for cell wall remodeling, which serve roles in the regulation of mechanical properties of the primary cell wall, wound healing, and plant morphogenesis [156].

According to nutrient availability and functions, the ECM of animals, plants and fungi differ in their relative content of proteins and carbohydrates. In the case of heterotrophic animals and fungi, nitrogen (a key element in proteins and nucleic acids) is not as limiting as in plants, for which this element is a nutritional bottleneck. Accordingly, the protein fraction is considerably higher in animal (20–35%; [133]) and fungal (30–50% in yeast and 20–30% in filamentous fungi; [61]) than in plant ECMs (2–10%; [159]).

The ECM also has a key function in cell–cell attachment for the establishment of multicellularity. Through aggregational adhesion, initially separated cells can firmly attach to each other either by secreting a viscoelastic and “sticky” ECM, or through the interaction of velcro-like surface proteins that “hook” cells together [39]. As these bonds can readily re-form after breaking, cells can rearrange, actuating a dynamic multicellular structure with rich physics and biology. Permanent connections/links, on the other hand, can be formed via incomplete cell separation processes in which mother and daughter cells remain at least partially physically attached. These connections, however, can generally not reform once broken. All three processes are common in nature and observed in land plants, algae, fungi, and in some stages of animal development [39, 104]. The huge variety of functional combinations of permanent and reformable intercellular bonds goes far beyond the scope of this review. Hence, the reader is referred to the mentioned literature for further details.

### Cell junctions

**Fig. 3:**
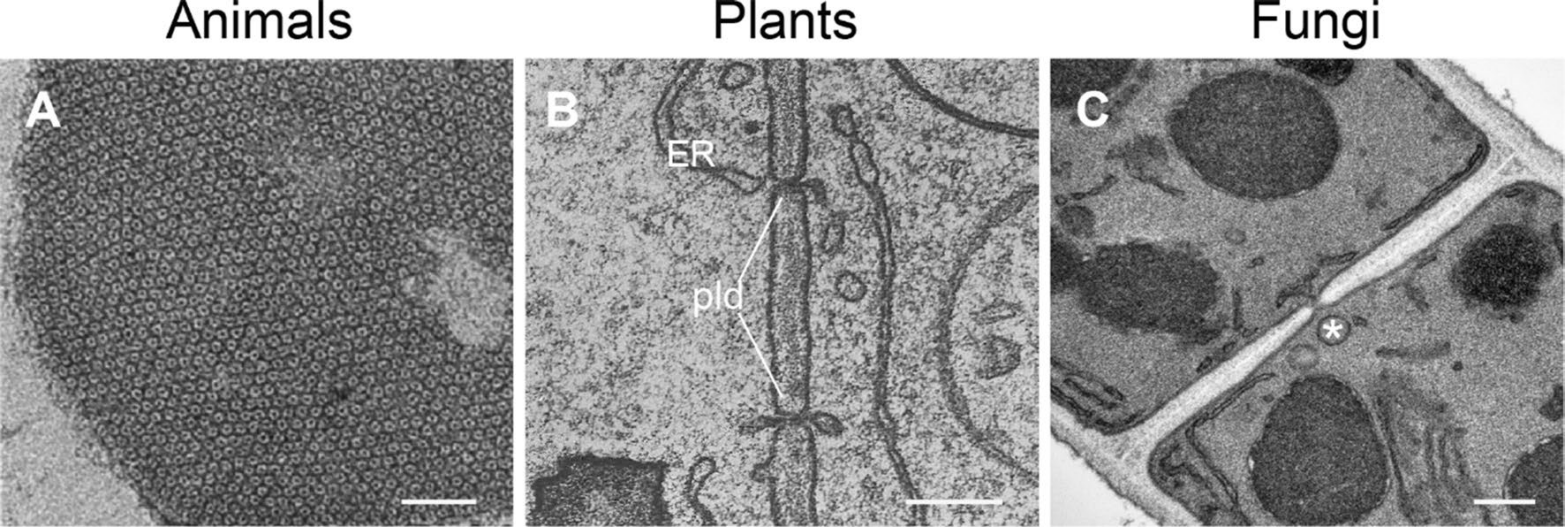
Transmission electron micrographs of cell–cell connections in animals, plants and fungi. **A** Cluster of an isolated junctional plaque from rat liver showing the gap junctions in a transversal cut. Adapted with permission from Ref. [84]. Scale bar 0.05 µm. **B** Plasmodesmata (pld) in a maize (*Zea mays*) root tip, with the endoplasmic reticulum (ER) spanning through the cell wall and linking neighboring cells. Adapted with permission from Ref. [124]. Scale bar, 0.1 µm. **C** Porous septum between two supracellular compartments of a *Zymoseptoria tritici* hypha. Note the Woronin body (*) (see Sect. “Organelles”) positioned right in front of the septal pore. Courtesy of Gero Steinberg, University of Exeter, U.K. Scale bar, 0.2 µm

Cell junctions, sometimes also referred to as intercellular bridges, provide in animals elaborate contacts or adhesion sites between neighboring cells and connect them to the underlying basal lamina, a layer of ECM. Cell junctions are especially abundant in endothelial and epithelial tissues and maintain in the latter the paracellular barrier. In vertebrates, adherens junctions, focal adhesions, desmosomes and hemidesmosomes mainly function in connecting cells with each other or the ECM, while tight or occluding junctions act as barriers between cells to restrict the movement of water [60]. Invertebrates have several other types of specific junctions, for example septate junctions in *Drosophila* or apical junctions in *C. elegans* [105]. In addition to these anchoring junctions, which support cohesion of tissues, channel-forming junctions such as gap junctions rather function in cell–cell communication by connecting the cytoplasm of neighboring cells exchanging metabolites, signaling molecules including RNAs, and even electrical impulses as in heart cells (Fig. 3A). High cytosolic Ca^2+^ or proton concentrations, which serve as cellular danger signals, promote the closure of gap junctions.

Due to their surrounding cell walls, adjacent plant and fungal cells often lack the need for anchoring junctions. A diffusion barrier, however, that is functionally analogous to the tight junctions of animal cells is found in plant roots in the form of the Casparian strip. The radial and transverse walls of root endodermal cells contain water-impermeable impregnations that prohibit the free diffusion of solutes in the extracellular space and that enforce regulated transport through the cell [63].

Similar to the gap junctions in animals (Fig. 3A), in plants, gaps in the cell wall enable the formation of cytoplasm-filled bridges between cells in the form of plasmodesmata (Fig. 3B). However, in comparison to the narrow (~ 1.5–2 nm in diameter) gap junctions, which are created by channel-forming connexin proteins that only permit the passage of small molecules, plasmodesmata are much wider (~ 20–200 nm in diameter) and allow also the passage of complex macromolecules [179]. Plasmodesmata connect plant cells to a syncytium-like cytoplasmic continuum termed the symplast [53]. Interestingly, viral movement proteins are able to dilate plasmodesmata to facilitate their cell-to-cell distribution [204]. Conversely, plants can close plasmodesmata transiently or permanently by the local deposition of the β-1,3 glucan polymer callose [114].

Similar to plant cells, connections that allow passage of macromolecules and organelles between adjacent cells exist in the form of septal pores in filamentous fungi (Fig. 3C). These breach the internal cross walls of hyphae (septa) and enable cytoplasmic continuity [213]. The morphogenesis of septal pores is analogous to the formation of primary plasmodesmata, i.e. they leave an opening in a constricting cross wall [41]. However, they cannot create an opening where none was before, as secondary plasmodesmata can. To establish cytoplasmic continuity de novo, fungi employ cell–cell fusion [54]. Cytoplasmic continuity between adjacent hyphal compartments—or at earlier developmental stages within germling networks—can be intermitted through septal pore closure with Woronin bodies (see Sect. “Organelles”) and subsequent consolidation of the seal [197].

For animal cells, tunneling or membrane nanotubes [178], protrusions that extend from the plasma membrane and touch other cells over long distances, have been also suggested to exchange metabolites, signals and genetic material, and even organelles and pathogens. However, their presence and function remains controversially discussed [236]. These and other aspects are further elaborated in a cross-kingdom comparative review that provides additional insights into intercellular connections in plants, animals and fungi [19].

### Organelles

Most membrane-bound organelles are present in the majority of eukaryotic cells. Nonetheless, a few differences exist between the kingdoms of life. As autotrophic, photosynthetically active organisms, plants possess chloroplasts—semiautonomous organelles whose main task is the conversion of light energy into chemically bound energy mostly in the form of carbohydrates. Chloroplasts are the most prominent members of a heterogeneous family of plant organelles, the plastids. Other members of this family comprise for example chromoplasts and amyloplasts, which serve roles in pigment and starch storage, respectively. Chloroplasts and other plastid types differentiate from an undifferentiated progenitor organelle, the proplastid. According to the endosymbiont hypothesis, plastids derived from photosynthetically active cyanobacteria that were captured by a basal eukaryotic cell lineage approx. 1.5 billion years ago, most likely via an endocytic process. Like semiautonomous mitochondria, plastids are covered by a double membrane and have their own circular genomes and protein biosynthesis machinery. Yet, most of their proteins are encoded by the nucleus and need to be imported post-translationally into the organelle [89]. A distinctive feature of chloroplasts is the presence of internal interconnected membrane sacks, the thylakoids. Like the multifold invaginated inner mitochondrial membrane, forming the cristae that harbor the respiratory chain components, the thylakoid membranes accommodate the components of the photosynthetic electron transport chain. Both structures, cristae and thylakoids, enlarge considerably the organellar membrane surface area. It has been proposed that these membrane enlargements originate from the bacterial progenitors that according to the endosymbiont theory gave rise to chloroplasts and mitochondria (cyanobacteria and α-proteobacteria, respectively [142, 218].

A further difference is the existence of vacuoles in plants and fungal cells. These organelles are functionally in part equivalent to the lysosomes of animal cells (which usually occur in higher numbers), yet acquired additional tasks in the course of evolution. In plant cells, the vacuole is as the lysosome in animals a lytic compartment, but also serves as a storing site for ions, (toxic) metabolites, (storage) proteins, and pigments. It further sequesters heavy metals and xenobiotics and plays a major role in cellular osmoregulation by establishing the turgor pressure, which is key to keep the body of herbaceous (nonwoody) plants upright [127]. Fungal vacuoles fulfill similar tasks but show a wide variety of architectures and roles in different species and in different cell types—their morphology and dynamics reflecting their ecological specialization [103, 162, 216]. Both plant and fungal vacuoles are, as the metazoan lysosomes, integral components of the secretory and endocytic pathways and are connected to other compartments of the endomembrane system via vesicle transport. This overlap is exemplified by the function of both lysosomes and vacuoles in autophagy. In animals, plant and fungi alike, autophagy is regulated through the interplay of cellular stress, ROS (reactive oxygen species), and TOR (target of rapamycin) signaling pathways [155, 220], including a highly conserved core machinery of ATG (autophagy-related gene-encoded) proteins [3, 111].

**Fig. 4:**
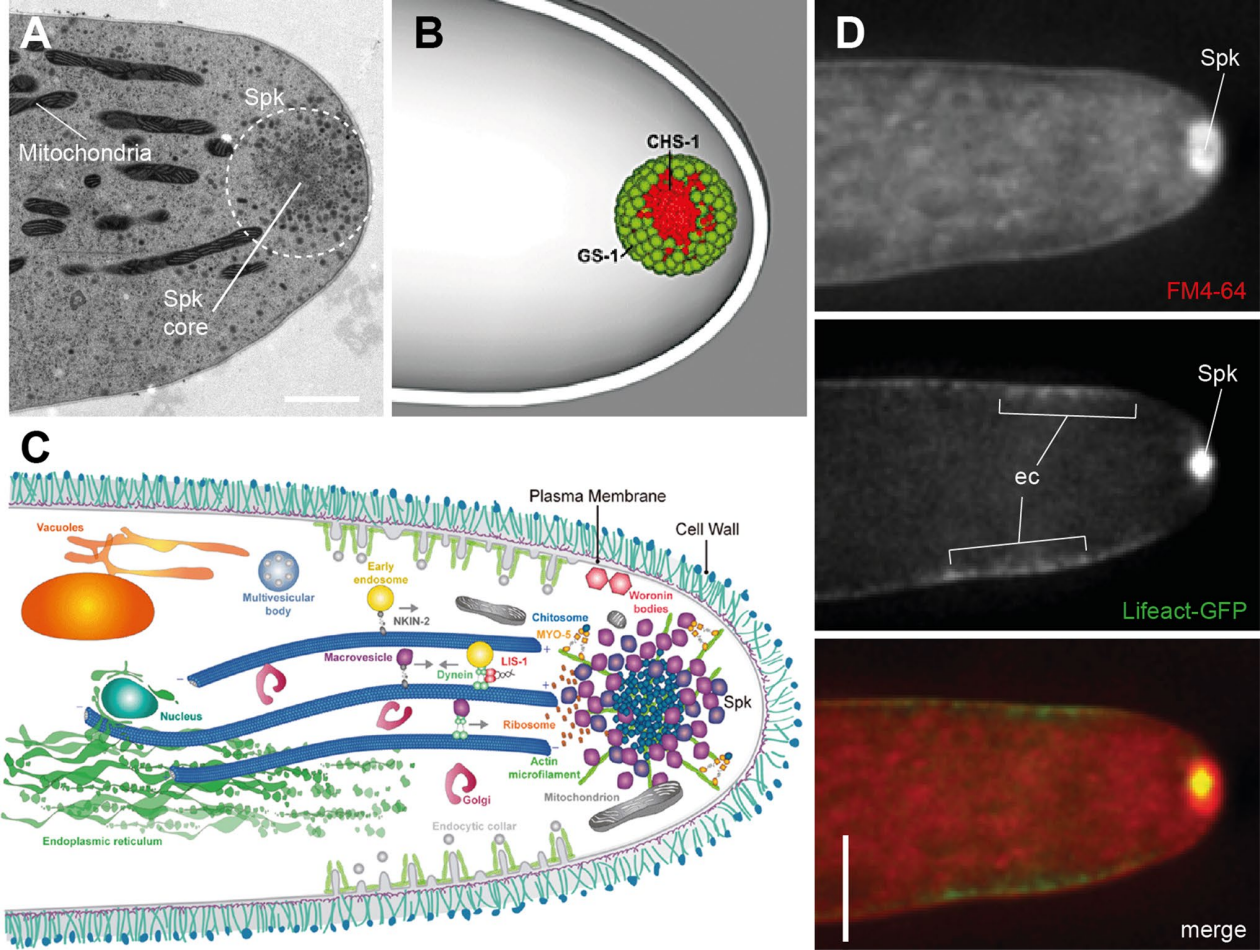
The fungal Spitzenkörper serves as an organizing center for hyphal tip growth. **A** A transmission electron micrograph section of the hyphal tip apex reveals the Spitzenkörper (Spk) as a dense vesicle cluster comprising a core with a surrounding vesicle cloud. Mitochondria are recruited into the apex to provide the huge amounts of energy required for fast tip growth. Image reproduced with permission from Ref. [169]. Scale bar, 500 nm. **B** The three-dimensional model of the functionally stratified Spk shows the glucane and chitin synthases GS-1 and CHS-1, respectively, as one of its main constituents (reproduced with permission from Ref. [214]). **C** Simplified schematic representation of the highly complex fungal tip growth machinery in which the Spk acts as a vesicle relay station: secretory vesicles deliver building blocks for plasma membrane and cell wall biosynthesis along microtubules towards the Spk. From there, vesicles are guided along F-actin tracks for targeted exocytosis to the apical plasma membrane to drive polarized hyphal tip extension. Not-incorporated material becomes reused through a subapical endocytic collar allowing for the very high tip extension rates seen in fungi (reproduced with permission from Ref. [165]). **D** Co-imaging of the fluorescent membrane marker FM4-64 with the F-actin reporter Lifeact-GFP shows the close functional relationship between exo- and endocytosis dynamics (*ec* endocytic collar) with the actin cytoskeleton (reproduced with permission from Ref. [14]). Scale bar, 5 µm. Please find further details on the topic in [62]

Fungal hyphae extend through highly localized secretion of cell wall material at the tip. In most filamentous fungi, this is controlled by the Spitzenkörper [131], a vesicle supply center that receives secretory vesicles from microtubules and sorts the cargo onto F-actin cables towards the plasma membrane (Fig. 4). Notably, in germ tubes—which lack a Spitzenkörper—a highly dynamic apical F-actin network is sufficient to distribute secretory vesicles within the expanding tip [14]. The formation of a Spitzenkörper marks the transition from germ tube to mature hypha [4]. The exceptionally high tip growth rates of fungal hyphae (averaging at around 1 µm/min across species) are facilitated by fast exocytosis via the Spitzenkörper and recycling endocytosis through a subapical F-actin collar [13, 205]. A comprehensive summary of the polarized tip growth apparatus in filamentous fungi has been provided elsewhere [167].

Fungi further possess specialized, peroxisome derived organelles, the Woronin bodies [95]. They usually reside near septa in filamentous ascomycete fungi (Fig. 3C). Their major role is to plug septal pores upon hyphal wounding to avoid leakage of the cytoplasm [128, 197]. Woronin bodies play roles in pathogenesis, such as the development and proper functioning of appressoria, specialized cells used by the rice blast fungus *Magnaporthe oryzae* to infect host plants [195], as well as for the formation of trapping rings in the nematophagous fungus *Arthrobotrys oligospora* [119].

Organelles rather specific for animals are melanosomes, acrosomes and phagosomes. Melanosomes are sites of synthesis, storage and transport of melanin, an abundant light absorbing pigment in animals, and are lysosome-related organelles derived from endosomes and/or lysosomes [221]. In mammals, they are found in skin and choroidal melanocytes as well as retinal pigment epithelial cells in the eye. In lower vertebrates, specialized pigment-containing cells called melanophores harbor melanosomes. Here, reversible aggregation and dispersion of these pigment granules permits fast color changes important for camouflage and communication. The acrosome is an acidic organelle in sperm cells of many animals including humans. It contains hydrolytic enzymes to help the sperm penetrating the egg’s coat during fertilization. It is a matter of debate whether acrosomes are lysosome-related or rather Golgi-derived organelles [102]. Phagosomes are vesicles formed around particles engulfed during phagocytosis [1]. While phagocytosis is used for food and nutrient uptake in unicellular organisms, such as *Dictyostelium discoideum*, in multicellular animals it removes microbes, apoptotic or senescent cells, and cellular or foreign debris. Thus, although related to endocytosis, the engulfment of large particles produces phagosomes, a type of organelle several orders of magnitude larger than endosomes. While phagosomes are prevalent in specialized cells like macrophages, neutrophils, and dendritic cells, many animal cells have some phagocytic capacity. For example, thyroid and bladder epithelial cells engulf erythrocytes, and retinal epithelial cells take up retinal rods.

### Membrane lipids

The lipid composition of the plasma membrane of animal, plant and fungal cells is largely similar and comprises phospholipids and sterols as the main constituents. The identity of the phospholipids is largely conserved in the three kingdoms of life. However, a subclass of phospholipids specific to animals (and bacteria) are plasmalogens. Differing from conventional phospholipids that have two ester-bound hydrocarbon chains, plasmalogens contain a vinyl-ether and an ester bond at the sn-1 and sn-2 positions, respectively, in the glycerol backbone. They constitute up to 10% of the total mass of phospholipids in humans (especially in the brain and in muscles). Another exception are the phosphoinositides (PtdIns), a group of phospholipids that contain phosphorylated versions of the cyclic polyol myo-inositol as head group. The inositol head group harbors five additional hydroxyl groups of which three (in positions 3, 4 and 5) can be dynamically (de-)phosphorylated by cytoplasmic lipid kinases and phosphatases. The resulting PtdIns can act as specific binding platforms for cytoplasmic proteins. While animal cells produce all seven possible mono-, di- and triphosphorylated versions, plant and fungal cells seem to lack the triphosphorylated PtdIns(3,4,5)P_3_ [91]. In addition, the occurrence of PtdIns(3,4)P_2_ in plant cells has been questioned [79].

Likewise, also the identity of the sterols differs between the three kingdoms of life. Cholesterol and ergosterol are the sole major sterols in animal and fungal plasma membranes, respectively. While cholesterol is the main sterol in the membranes and lipid droplets of animal cells [129]—with *C. elegans* as a well-known exception [110]—ergosterol is the main component to modulate the fluidity, permeability and integrity of the fungal plasma membrane [222]. By contrast, plant plasma membranes comprise a greater variety of (phyto)sterols, including complex mixtures, in which sitosterol, stigmasterol, 24-methylcholesterol and camposterol often prevail [76, 194]. Sterylglycosides, the sugar derivatives of membrane-bound sterols, are as well asymmetrically distributed amongst the three eukaryotic kingdoms. While sterylglycosides are common in plants and fungi, they are rarely found in animals [71]. The ratio between free and glycosylated sterols alters the biophysical properties of the membrane considerably and, thus, provides functional control over sterol-enriched lipid microdomains [72].

The presence of photosynthetic organelles has profound influence on the overall lipid content of and flow within plant cells [186]. Fatty acid synthesis occurs other than in animals and fungi not in the cytosol but in the chloroplast and other plastids. The chloroplast membranes contain substantial levels of glycosyldiacylglycerols, which overall can make up more than 70% of chloroplast lipids, especially when phosphorus supply is limited [88]. Glycosyldiacylglycerols share a common 1,2-diacyl-glycerol backbone with phospholipids, but a carbohydrate rather than phosphate occupies the third sn position. In particular, mono- and digalactosyldiacylglycerols as well as sulfoquinovosyldiacylglycerols, whose head group is a modified sulfate deoxyglucose, are main components of the chloroplast thylakoid membranes. Accordingly, galactosyldiacylglycerols can make up the largest lipid fraction of leaves while they are only found in traces in animals [193]. Galactolipids are integral components of photosynthetic protein complexes, which are embedded into the thylakoid membrane, and required for their functionality. In addition, they contribute to protect the photosynthetic machinery in high light conditions [5, 88].

### Cytoskeleton

The cytoskeleton is an extended network composed of different types of linear and branched proteinaceous polymers that serves numerous purposes. These include providing shape for cells, enabling their movement, mediating intracellular transport, and supporting chromatin segregation and cell division during mitosis and cytokinesis. While actin filaments and microtubules are present in all three phyla, the situation is different for intermediate filaments and septins, the latter being often considered the fourth component of the cytoskeleton [137]. Furthermore, centriole-containing centrosomes, which serve as microtubule-organizing centers (MTOCs) in metazoa, are absent from plants and fungi.

Intermediate filaments of metazoan cells constitute a family of related fibrous proteins that assemble in a characteristic non-polar manner into filament networks. One of their main tasks is to provide physical support to cells [83]. In humans, more than 70 types of intermediate filament proteins exist. Prominent members comprise keratins, lamins and desmins. While most intermediate filaments are cytoplasmic, the lamins line the inner side of the nuclear envelope as a lamina meshwork that anchors (hetero-)chromatin to the nuclear periphery, which modulates gene expression [81]. The lamina is connected to the different cytoskeletal structures via the LINC (linker of the nucleoskeleton and cytoskeleton) complexes, which are formed by membrane proteins residing in the nuclear envelope. This is important for positioning the nucleus within cells but also for mechanotransduction [93, 115]. Based on the analysis of fully sequenced genomes, orthologs of genes encoding canonical intermediate filament proteins known from metazoan species are lacking in plants and fungi [12, 82]. It has been suggested that intermediate filaments might be dispensable for cells that are covered by a stiff cell wall, alleviating the need for structural support provided by cytoskeletal elements [83]. However, the existence of several analogous proteins with equivalent functions has been proposed, particularly with regard to nuclear lamins, especially in plants [35, 36, 90, 202, 211] and, less though, in fungi [67, 106]. In particular the plant CROWDED NUCLEI (CRWN) proteins might play similar roles as metazoan lamins in regulating chromatin organization and gene expression [90, 180].

Septins are a group of GTP-binding proteins that assemble into short homo- and heteromers and ring-like structures [228]. They play a canonical role in cell division as reflected by their name, which relates to septa formation during budding yeast cytokinesis [27]. However, they also participate in numerous other processes such as the formation of submembrane networks to limit the lateral diffusion of membrane proteins [52] or the regulation of cell polarity and cell–cell fusion [15]. Septins are present in most eukaryotes. However, they are apparently lacking in land plants, although they can be found in their sister lineage, the green algae (chlorophytes). Phylogenetic analyses suggest that septins have evolved from a common eukaryotic ancestor, distributed and diverged within the eukaryotes, and were lost in some lineages like the land plants [145, 230].

**Fig. 5:**
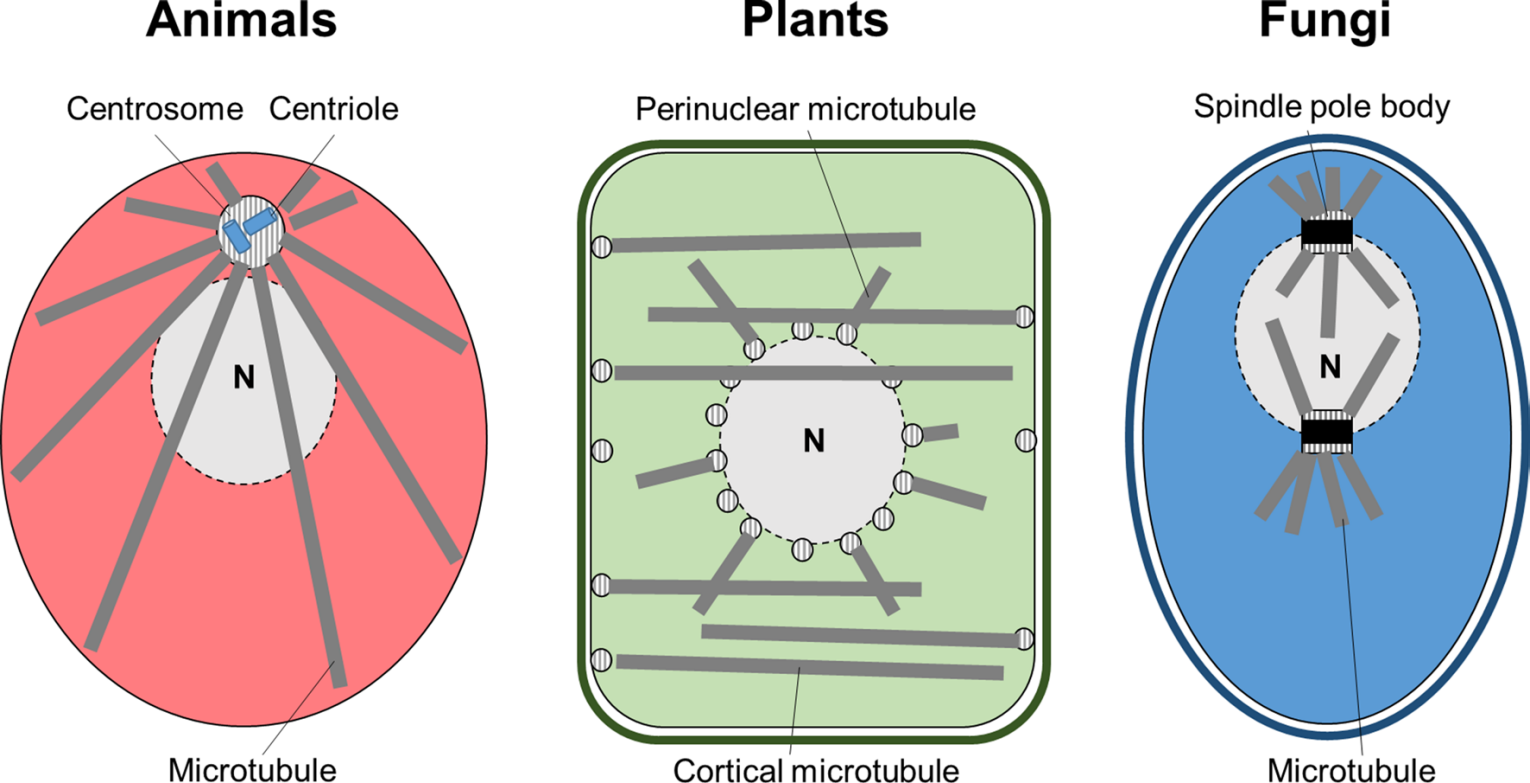
Modes of microtubule nucleation in animals, plants and fungi. The Figure schematically illustrates common types of microtubule nucleation in multicellular eukaryotes. In animals, the centrosome with its two enclosed centrioles often serves as the center of microtubule nucleation. It is frequently located near the nucleus (N). In plants, microtubules can be nucleated at various sites on the nucleus, giving rise to perinuclear microtubules, or at the plasma membrane, yielding cortical microtubules. Fungi possess spindle pole bodies for the nucleation of microtubules. Spindle pole bodies are often embedded in the nuclear envelope. The scheme was inspired by a similar Figure published previously [187]

Centrosomes are cellular structures that serve as the MTOCs for proliferating animal cells and play a key role during mitosis by orchestrating the spindle apparatus. They harbor two perpendicularly oriented centrioles—special barrel-shaped microtubular structures—that are embedded in a protein matrix termed the pericentriolar material (Fig. 5). Interestingly, as basal bodies, centrioles also organize primary cilia [224]. Similar to septins, the centrosome is thought to have evolved in an ancestral eukaryote. The latter, however, was structurally modified to a degree that bona fide centrioles are generally regarded as being lost in the plant and fungal lineages [32]. Interestingly, centrioles are also lacking in planarians [8]. In acentrosomal phyla, microtubules emerge from different types of MTOCs. In plants, flexible MTOCs without centrioles can arise on the plasma membrane, the nuclear envelope, and even organelles in dependence of the cell type and organism and/or the physiological conditions [116] (Fig. 5). Fungi evolved a centriole-less MTOC known as the spindle pole body [143]. Spindle pole bodies are large, proteinaceous, disc-shaped structures that lack centrioles and are either continuously embedded in the nuclear envelope or become inserted into the nuclear envelope prior to mitosis (Fig. 5) [94]. While spindle pole bodies and centrosomes are morphologically distinct, both organelles share components and regulators. Analysis of fungal spindle pole bodies have identified some of the most conserved and important MTOC components, including γ-tubulin, first identified in the filamentous fungus *Aspergillus nidulans* [146]. Apart from spindle pole bodies, transient MTOCs in the division plane, nuclear-envelope associated MTOCs in interphase cells as well as septum-associated MTOCs may occur in fungi [234].

Interestingly, while centrosomes are often considered as the MTOCs in animals, upon differentiation, many cells organize their microtubules at non-centrosome sites. For example, in striated muscle cells at the nuclear envelope [203], or in epithelial cells at the apical plasma membrane and the Golgi apparatus [9].

### Motor proteins

Motor proteins is the collective term for a class of proteins that use adenosine triphosphate (ATP)-driven mechanochemical cycles to move along polar cytoskeletal elements (actin filaments and microtubules). The generated mechanical force can be used either to carry cargos (e.g. vesicles, organelles, proteins, RNA, or chromosomes), or to shift cytoskeletal elements (e.g. during muscle contraction and in contractile stress fibers or microtubule sliding within the mitotic spindle). Three types of motor proteins are generally present in eukaryotes. They comprise the myosins, which move towards the plus end of actin tracks, and the kinesins and dyneins, which generally step in opposite directions (towards the plus and minus end, respectively) on microtubules. However, exceptions such as minus-end-directed myosins and plus-end-directed kinesins exist. Axonemal dynein, found in eukaryotic cilia and flagella, is crucial to cell motility and fluid transport, as discussed above (see Sect. “﻿Motile cilia and flagella”﻿). While myosins and kinesins can be found in all eukaryotes, including animals, plants and fungi, dyneins are only present in animals and fungi but absent from plants [226]. Dyneins are large, typically minus‐end‐directed multi-subunit motors that exist as cytoplasmic and axonemal forms, of which the latter have dedicated functions in flagellar activity. Each dynein is composed of at least one dynein heavy chain in combination with a variable number of intermediate chains, light intermediate chains, and light chains. Higher plants lack cytoplasmic dyneins and gradually lost axonemal heavy chain and intermediate chains in the course of evolution, likely as a consequence of a shift from flagellated to non-flagellated sperm cells [123], while the respective light chains may have undergone neofunctionalization in this lineage [31]. To compensate for the loss of transport towards the minus end, the plant kinesin-14 subfamily expanded and evolved members that enable minus-end-directed transport [229].

**Table 1:** Cross-kingdom comparison of cell biological features in animals, plants and fungi

	Animals (metazoa)	Plants (Viridiplantae)	(Filamentous) Fungi
**Cell features**			
Cell size and shape	Smaller cells, irregular structures common	Larger cells, often spherical or cubic	Larger cells, typically tube-shaped hyphae
Protrusions	Cellular protrusions such as microvilli, pseudopods and cilia (undulipodia) present	Cellular protrusions such as root hairs present; cilia only in mosses and ferns—not in seed plants	Cellular protrusions, mainly as germ tubes and conidial anastomosis tubes
Flagellae	Present on sperm cells	Flagellated sperm cells in some phyla (bryophytes, ferns and some gymnosperms)	Mostly absent (apart from basal lineages)
Motile cilia	Present	Absent	Absent
Totipotency	Rare	Common	Common
**Extracellular matrix**			
Composition	Soft network of macromolecules, composed of glycoproteins and glucosaminoglycans with little polysaccharides (hyaluronic acid)	Solid cell wall mainly composed of carbohydrate polymers (lead molecule cellulose), plasmolysis possible, occurrence of more plastic primary and very rigid lignified secondary cell walls	Semi-solid cell wall matrix composed of carbohydrate polymers (lead molecules chitin and glucans) cross-linked with glycoproteins
**Cell junctions**			
Types	Focal adhesions, desmososmes, hemidesmosomes, gap junctions, tight junctions	Plasmodesmata, Casparian strip	Germling and hyphal fusion, porous septa
**Organelles**			
Specific organelles	None (?)	Plastids (chloroplasts)	Woronin bodies, Spitzenkörper
Lysosomes	Many small lysosomes	Large central vacuole	Functionally diverse vacuoles
Golgi apparatus	Few stacks	Many stacks	Tubules and sheets, sometimes stacked
Active transport	Along microtubules	Along actin filaments	Along microtubules and actin filaments
Cytoplasmic streaming	Rare (oocytes)	Common	Common
**Lipids**			
Sterols	Cholesterol	Different (phyto-)sterols	Ergosterol
Phosphoinositides	All seven possible mono-, di- and triphosphorylated PtdIns versions are present	PtdIns(3,4)P_2_ and PtdIns(3,4,5)P_3_ so far not reliably identified	PtdIns(3,4,5)P_3_, so far reliably identified
**Cytoskeleton**			
Centrioles	Present	Absent	Absent
Microtubule nucleation	Typically at centrioles, but also non-centrosomal MTOCs exist	At various sites on the nuclear envelope or at the plasma membrane	At spindle pole bodies
Motor proteins	Myosins, kinesins and dyneins	Myosins and kinesins (no dyneins but instead minus-end oriented kinesins)	Myosins, kinesins and dyneins
Septins	Present	Absent	Present
Intermediate filaments	Present (including lamins)	Absent, but possibly proteins with functions analogous to lamins present	Absent, but possibly proteins with functions analogous to lamins present
**Motor proteins**			
Dyneins	Present	Absent	Present
**Cell cycle regulation**			
p53	Present	Absent	Present („p53-like “)
Rb	Present	Present	Absent
**Mitosis and cytokinesis**			
Mitosis	Open	Open	Closed or semi-open/semi-closed
Cytokinesis	Contractile actomyosin ring	New cell wall (phragmoplast, cell plate)	Contractile actomyosin ring
**Apoptosis**			
Caspases	Present	Absent (but so-called metacaspases present)	Absent (but so-called metacaspases present)
Bax/Bax Inhibitor	Present/present	Absent/present	Absent/present
**Signal transduction**			
GPCRs	Many GPCRs	No (known) GPCRs but heterotrimeric G protein	Few GPCRs (e.g. pheromone response, nutrient sensing, cAMP)
Calcium channels	Many different types	Fewer types	Numerous different types
**Growth**			
Tip growth	Only in neurons	Few cell types	Common (for apical extension of hyphae)
**Multicellular context**			
Mobile cells	Present	Absent	Absent
Contractile cells	Present	Absent	Absent
Electrically excitable cells	Nerve and muscle cells	Guard cells	Galvanotropism
Long distance transport	Via circular system	Via transport tissues (phloem and xylem), no cell transport	Long-distance endosome trafficking
Tumor formation	Frequent, including metastases	Rare, no metastases	No
Non-self recognition (immunity)	Innate and adaptive immunity, professional immune cells and cell-autonomous immunity	Innate immunity (RLK- and NLR-based), cell-autonomous immunity	Innate immunity (NLR-based)

## Cellular functions

### Active intracellular transport

Passive intracellular transport via random motion (diffusion) is only efficient on short distances and for small cargos. Thus, compartmentalized eukaryotic cells rely additionally on active transport, which is driven by motor proteins along cytoskeletal elements. Active intracellular transport processes in animal cells are based on microtubules in combination with kinesin and dynein motors for long-range movement and actin filaments together with myosins for short-range transport. This generally applies to a broad variety of organelles and vesicle trafficking [10]. By contrast, all major organelles are shuttled along actin filaments with the help of myosin motors in plant cells, while kinesins mediate short-range positioning along microtubules [25]. Active intracellular transport along cytoskeletal tracks in fungi is organized equivalent to the animal system [167].

As outlined before (see Sect. “Cell size and shape”), plant and fungal cells are typically larger than animal cells and often possess extensive vacuole systems (see Sect. “Organelles”). In these taxa, organelles and other cellular content constantly move around a central vacuole (plants) or a system of vacuoles (fungi). This process is called cytoplasmic streaming. In plants, it is driven by motor proteins that travel along cytoskeletal elements [206], while in fungal cells it is based on osmotic pressure differences [154]. It is believed that cytoplasmic streaming compensates for the larger size and steric conditions of plant and fungal cells, which limit diffusional processes [206]. Notably, cytoplasmic streaming is accordingly also described in the large oocytes of several animal species, arguably best studied in *Drosophila* oocytes [158].

### Long distance transport

Long distance transport within animal bodies can be mediated via circulatory systems. These can be a closed tubular circuit, such as the blood circulatory system in vertebrates/homeotherms, but also an open system, such as the insects’ hemolymph system. Both have the capacity to transport signaling molecules, nutrients, and a variety of different cell types. Plants and fungi lack elaborate circulatory transport conduits. Although plants have two major systems for long distance transport, the xylem (for upstream transport of water and minerals) and the phloem (for downstream transport of assimilates), these differ in several aspects from the animal circulatory systems. First, the two pathways are separate from each other and do not form a closed circuit as the blood stream. Second, the tube diameter is restricted to the width of single cells, preventing cellular transport via these routes. In fungi, the only option for long distance transport is through the hyphal tube and is, therefore, also restricted by the inner tube diameter. Nevertheless, profuse branching can rapidly establish numerous parallel tubes to increase net flow rates, and increased vegetative hyphal fusion rapidly expands the interconnected fungal colony network for faster and more efficient cargo distribution [77]. Some species even establish high-conductivity cords, so called rhizomorphs, to cover long distances on nutrient-poor substrate, such as soil [29]. The driving force of long-distance transport in fungi is the mass flow of water. Filamentous fungal colonies form integrated hydraulic systems, in which the increase in volume at the expanding hyphal tips at the fungal colony edge requires either an equivalent water uptake or volume reduction in another region of the mycelium [78]. The localized regulation of cytoplasmic streaming (see Sect. “Active intracellular transport”) is thus important for targeted distribution of water, nutrients and organelles to specific regions of the fungal colony. The distribution of nuclei throughout the hyphal network provides a highly vivid example for this phenomenon [171].

### Cell cycle regulation

The core machinery of cell cycle regulation, including cyclins and cyclin-dependent kinases, is conserved among eukaryotes. However, there exist lineage-specific differences regarding proteins that fine-tune cell cycle regulation. The human proteins p53 and Rb (retinoblastoma) are key negative regulators of the cell cycle and both serve as “tumor suppressors” by preventing inadequate cell divisions. The transcription factor p53 is activated upon DNA damage to govern DNA repair, mediate cell cycle arrest, and, if necessary, trigger apoptosis. Plants lack a recognizable p53 ortholog. Instead, they possess a transcriptional regulator (SOG1) that fulfills similar tasks [231]. Two members of the “p53-like” superfamily of proteins (Ndt80 and CSL) are widespread in fungi where they play a role in nutrient sensing, suggesting that this could be the ancestral function of p53-like proteins [99]. The human Rb protein negatively regulates cell cycle progression through its interaction with members of the E2F/DP family of transcription factors. Contrary to p53, Rb and its mode of action via interaction with E2F transcription factors is evolutionarily conserved in plants [42] but not in fungi [75].

### Mitosis and cytokinesis

**Fig. 6:**
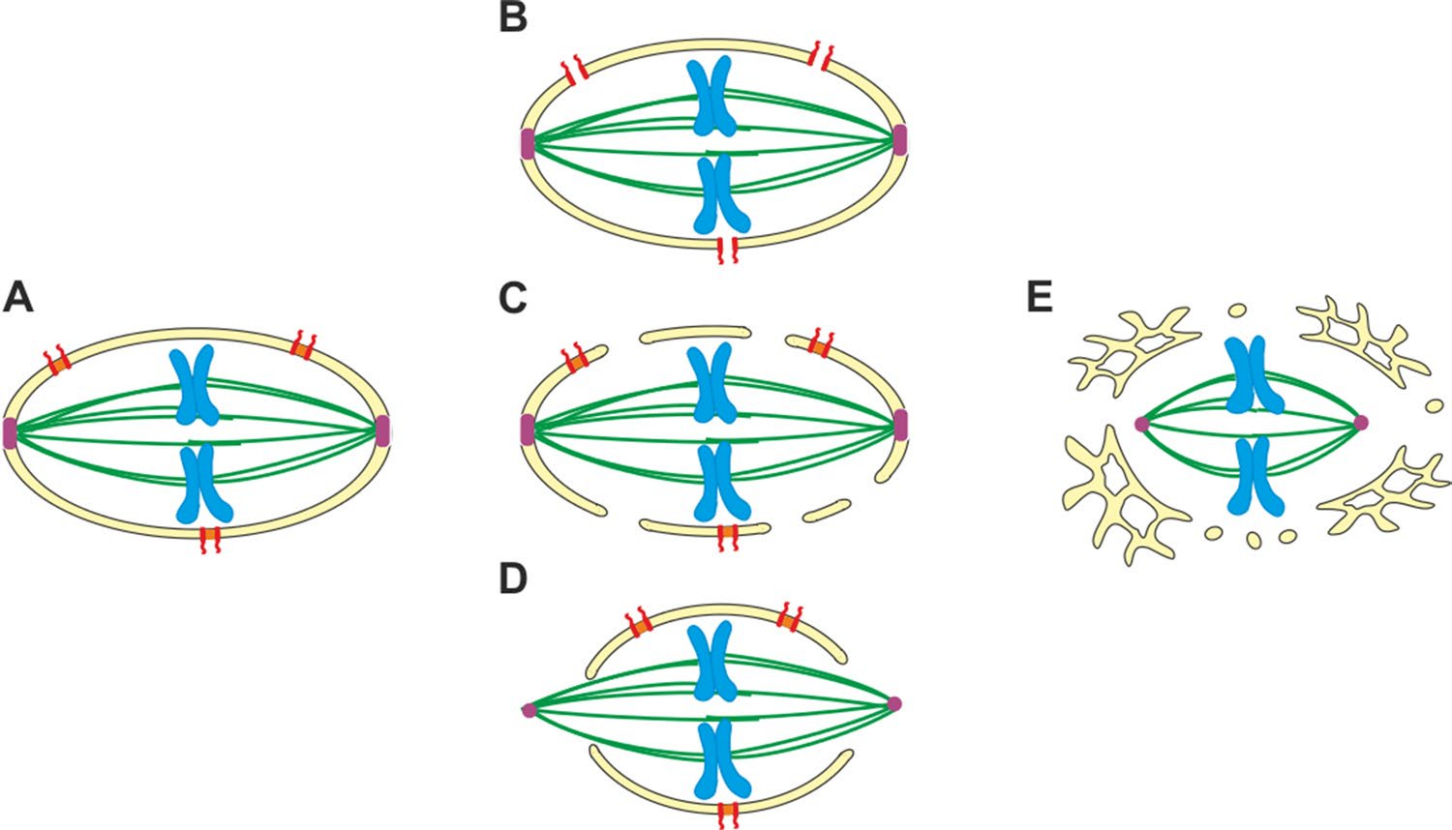
Different strategies for mitosis deployed in eukaryotes. **A** During closed mitosis, often observed in fungi, the mitotic spindle assembles within the nucleus. Spindle pole bodies (pink; see also Fig. 5) are integrated within the nuclear envelope (yellow) and nucleate microtubules (green) that attach and segregate the duplicated chromatids (blue) within the boundaries of an intact nuclear envelope. Nuclear pore complexes (red–orange) integrated in the nuclear envelope remain intact and functional during this process. **B–D** Some types of mitosis are neither strictly closed nor strictly open. In the filamentous fungus *Aspergillus nidulans*, but also female germline stem cells of *Drosophila*, the nuclear envelope remains intact during mitosis, but nuclear pore complexes partially disassemble so that tubulin monomers diffuse into the nucleus and polymerize to microtubules (**B**). By contrast, entry of *Drosophila* early embryos into mitosis is accompanied by a partial breakdown of the nuclear envelope, but with large fractions of the nuclear envelope including nuclear pore complexes remaining intact (**C**). In many organisms and cell types, as e.g. in *Caenorhabditis elegans* early embryos, upon entry into mitosis polar openings form in the nuclear envelope, which allow microtubules nucleated from cytoplasmic centrosomes to reach, attach and segregate the duplicated chromatids (**D**). **E** During open mitosis seen in many vertebrate cells and in plants, the nuclear envelope including nuclear pore complexes dis- and reassemble during mitosis to allow the formation of a cytosolic spindle apparatus formed by centrosome-nucleated microtubules (see also Fig. 5)

Mitosis is the part of the eukaryotic cell cycle during which the replicated chromosomes of dividing cells are separated into two new nuclei. It is subdivided into different phases (pro-, prometa-, meta-, ana- and telophase), of which anaphase refers to the period during which the replicated sister chromatids separate. For this process, metazoan and plant cells often fully disassemble their nuclear envelope and nuclear pore complexes allowing intermixing of the cytoplasm and nucleoplasm. This type is therefore referred to as “open mitosis”. By contrast, most fungal cells either keep their nuclear envelope fully intact (“closed mitosis”) or have a ruptured/fenestrated nuclear envelope during mitotic anaphase (“semi-open mitosis” or “semi-closed mitosis”) [20, 196] (Fig. 6), but also these distinctions are not absolute. For example, *Aspergillus nidulans* keeps the nuclear envelope during mitosis intact but due to a partial disassembly of nuclear pore complexes the separation between cytoplasm and nucleoplasm is lost [196] (Fig. 6B).

Semi-closed mitosis can also occur in metazoan cells, e.g. in *C. elegans* early embryos (Fig. 6D). Even within the same organism a spectrum of mitotic forms can exist: While *Drosophila* somatic cells undergo open mitosis (Fig. 6E), their female germline stem cells keep the nuclear envelope during mitosis intact but disassemble nuclear pore complexes [48] (Fig. 6C). Primordial germ cells show a more “open” mode with large nuclear envelope fenestrations, which is also observed during the syncytial embryonic divisions.

Multinucleate filamentous fungi display three types of mitotic patterns: synchronous, parasynchronous and asynchronous. In synchronous division, all nuclei within the same hyphal compartment divide simultaneously. In parasynchronous division, mitosis is initiated in one spot and then a wave of mitoses travels down the hyphae linearly so that nuclei divide sequentially, almost as dominos fall in a line. In asynchronous division, nuclei divide independently of neighbors, giving an apparent random spatial and temporal pattern to mitosis [65]. The molecular details of how cell cycle and gene expression of individual nuclei is regulated are still very poorly understood. Similarly, it is unclear whether and, if so how, nuclei within the multinucleate syncytium of the hyphal network cooperate or rather compete [134].

The actual cell division (cytokinesis), the separation of the cytoplasm following segregation of the chromosomes, often occurs with the final step of mitosis or meiosis. In animal cells, this involves reorganization of the mitotic spindle to a central spindle (or spindle midzone) with microtubule fibers bundled between the spindle poles. After specification of the division plane, an actin-myosin ring assembles and contracts to form a cleavage furrow. The furrow ingresses until a midbody, a microtubule-rich structure at the junction of the two dividing cells, is generated. Most animal cells remain then connected by an intercellular cytokinetic bridge for up to several hours until they finally split by an actin-independent process named abscission, which involves the removal of cytoskeletal structures from the cytokinetic bridge, constriction of the cell cortex, and plasma membrane fission mediated by the endosomal sorting complex required for transport (ESCRT) machinery.

The solid cell walls require a different mechanism in plant cells. An actin- and microtubule-rich barrel-shaped structure, the phragmoplast, is formed in the equatorial plane and enables the transport of new cell wall material in Golgi-derived vesicles towards the middle of the dividing plant cell. Here, a new cell wall is established upon soluble *N*-ethylmaleimide-sensitive-factor attachment receptor (SNARE) protein-mediated vesicle fusion events from inside to outside, initially as a disc termed the cell plate, which is extended to both sides until full cell separation is achieved [215].

Filamentous fungi accomplish cytokinesis similar to animal cells through the formation of a contractile ring that pulls the plasma membrane inwards. The cell wall biosynthesis machinery is dragged along and consolidates the spatial separation of the two daughter compartments. The dynamics of the pulling actomyosin tangle have impressively been demonstrated in *N. crassa* [41]. For the formation of porous septa, the constriction arrests before completion, whereas for the formation of closed septa or the separation of conidia the constriction proceeds until full closure. Individual conidia are furthermore fully separated from the chain by enzymatic degradation of the intervening cell wall in order to allow wind distribution. Elaborate signaling mechanisms known as the mitotic exit network and the septation initiation network coordinate the distribution of nuclei with compartmentalization of the hypha [219]. Further details on the molecular regulation of septation in filamentous fungi can be found elsewhere [138].

### Apoptosis

Apoptosis is a form of noninflammatory programmed cell death (PCD) in multicellular organisms that bears certain morphological and molecular hallmarks. It can be triggered by extrinsic and intrinsic factors and is associated with cell shrinkage, cytoplasmic blebbing, chromatin condensation, DNA fragmentation into discrete length units (DNA laddering), and ultimately the formation of apoptotic bodies [51]. A family of cysteine-dependent aspartate-specific proteases, the caspases, play a key role in the initiation and execution of apoptosis [101]. Apoptotic cell death is a decisive phenomenon during development and is implicated in the constant replacement of cells in the bodies of metazoa. PCD in the context of regular cell turnover is uncommon in plants and fungi, possibly since these organisms lack mobile phagocytic cells that could adsorb apoptotic corpses. Nonetheless, plants and fungi can undergo apoptosis-like PCD under certain conditions, like in aging, during reproduction, upon pathogen attack (plants), or protection of the genome against foreign DNA (fungi). Notable phenomena of the latter are heterokaryon incompatibility [118, 185] and spore killers [208]. Heterokaryon incompatibility describes the process in which hyphal compartments that have received genetically incompatible nuclei, e.g. after a hyphal fusion event [223], undergo PCD. They pass the apoptotic signal to their neighboring compartments to prevent spreading of unwanted genetic information throughout the mycelial network [66]. Spore killers are meiotic drive elements that cheat during sexual reproduction to increase their transmission into the next generation by destroying unwanted spores [232].

Parts of the core apoptotic machinery known from the animal kingdom are conserved in fungi. However, most of the respective protein families are reduced in size, and the network controlling PCD in fungi is less complex [189]. Apart from several apoptotic regulators, in particular *bona fide* caspases are lacking in fungi. Instead, fungi possess metacaspases, cysteine-dependent proteases that are arginine/lysine-specific and only distantly related to metazoan caspases [207]. Similar to fungi, apoptosis-like cell death occurs in plants during development as well as in response to environmental and biotic stress stimuli. Although plants, like fungi, lack many components of the canonical metazoan apoptotic machinery, several morphological and apoptotic features are nevertheless shared with animals. It was moreover suggested that the large central plant vacuole serves a role in the removal of cell death-related cell debris in the absence of a phagocytic system [43]. However, as in the case of fungi, plants have metacaspases instead of caspases [207]. Interestingly, despite the lack of prominent mammalian cell death regulators such as pro-apoptotic Bax and anti-apoptotic Bcl-2, both fungi and plants are responsive to these proteins and both also express a functional Bax inhibitor [43, 189]. Further details on the evolutionary origins of PCD pathways in the different kingdoms has recently been summarized elsewhere [87].

### Signal transduction

Although the general principles of signal transduction are similar in all three kingdoms of life, the details such as the types of ligands and receptors involved as well as the nature of the downstream signaling events differ. In addition, orthologous signaling components may be used in homologous or slightly different cellular contexts. Here, we can only cover the most prominent differences in signal transduction between animals, plants and fungi. These comprise distinctions in the types and numbers of cell surface receptors and some of the associated signal transduction components.

The family of membrane-resident heptahelical G-protein coupled receptors (GPCRs), whose members bind extracellular ligands and transmit the signal intracellularly via heterotrimeric GTP-binding proteins (G-proteins), composed of α-, β- and γ-subunit [172], is vastly expanded in animals. From simple members such as the worm *C. elegans* to complex mammals, animals encode typically hundreds of GPCRs, and these can represent up to 5% of the total number of genes [233]. In humans, there are approx. 800 GPCRs, half of which serve as olfactory receptors. The remaining receptors bind a broad variety of different proteinaceous and non-proteinaceous extracellular ligands. Most filamentous fungi express a considerably lower number of GPCRs and GPCR-like proteins. Depending on the species, between 36 and 76 different GPCRs and GPCR-like proteins grouped into 14 different classes have been identified and are involved in the sensing of sugars, amino acids, cellulose, cell wall components, sex pheromones, oxylipins, calcium ions, cyclic adenosine monophosphate (cAMP) or other ligands [28, 50, 108, 126]. Despite their reduced number in fungi as compared to animals, the downstream signal transduction wiring is equally complex and facilitates a multitude of cellular responses. It is still under debate whether plants possess any *bona fide* GPCRs [200]. Interestingly, however, plants have retained heterotrimeric G-protein subunits, which usually operate downstream of GPCRs, in the course of evolution. It is assumed that a regulator of G-protein signaling protein may control heterotrimeric G-protein activity in the absence of canonical GPCRs in (dicotyledonous) plants. Similar to GPCRs, this regulator is an integral membrane protein with seven membrane-spanning domains, and it promotes GTP hydrolysis (i.e., inactivation) of the heterotrimeric G-protein α-subunit, which is self-activating in plants [210].

Plant genomes code for expanded repertoires of plasma membrane-resident receptor-like kinases (RLKs), which typically comprise several hundred members. RLKs can harbor different kinds of extracellular domains, binding various ligand types, and have an intracellular serine/threonine kinase domain for downstream signaling [44]. They are implicated in a variety of physiological processes such as development, reproduction and immunity. At least some plant RLKs might operate through direct coupling to heterotrimeric G-proteins [120].

Another major class of cell surface receptors in animals are receptor tyrosine kinases (RTKs), with more than 50 different members in humans. Like plant RLKs, animal receptor tyrosine kinases can have a variety of extracellular domains. Upon ligand binding, they dimerize and trans-autophosphorylate each other via their cytoplasmic tyrosine kinase domains, which creates docking sites for downstream signaling components [117]. While tyrosine phosphorylation is common and widespread in animals, it is rare in plants, where serine/threonine phosphorylation prevails [188]. The latter is exemplified by the prominent role that RLKs play in various aspects of plant life.

Fungi are generally thought to lack tyrosine kinases, receptor tyrosine kinases and RLKs. Occasional publications, nevertheless, suggest the presence of a fungi-specific lineage of protein kinases that appears to be a sister group closely related to tyrosine kinases [235]. It remains, however, open whether this fungi-specific lineage of protein kinase comprises proteins with receptor function.

Calcium (Ca^2+^) ions are important second messengers that play a key role in various signal transduction cascades [16]. Cytoplasmic Ca^2+^ levels are generally kept low (in the nM or low µM range), with extracellular concentrations being 3–4 orders of magnitude higher (in the mM range). Plasma membrane-resident channels mediate the influx of Ca^2+^ ions upon their activation by upstream signaling components. In addition, some Ca^2+^ channels are located in membranes of internal Ca^2+^ stores such as the endoplasmic reticulum. In humans, six major classes of Ca^2+^ channels exist. These comprise the voltage-dependent Ca^2+^ channels, two-pore channels, inositol (1,4,5)-trisphosphate receptors, transient receptor potential channels, cyclic nucleotide gated channels and ionotropic glutamate receptor channels, of which two-pore channels and ionotropic glutamate receptor channels have the highest number of members (28 and 18, respectively). Plants do not have recognizable voltage-dependent Ca^2+^ channels, inositol (1,4,5)-trisphosphate receptors and two-pore channels but possess expanded cyclic nucleotide gated channel and ionotropic glutamate receptor channel families (20 members each in *A. thaliana*) [225], and recently were found to harbor a plant-specific family of Ca^2+^ channels [59]. Thus, consistent with a lower importance of electrical signals (see Sect. “Electrically excitable cells”) and the likely absence of inositol (1,4,5)-trisphosphate (see Sect. “Membrane lipids”), plants harbor a reduced repertoire of Ca^2+^ channels as compared to animals.

Ca^2+^ signaling is involved in diverse cellular processes also in fungi and employs conserved signaling components similar to the regulatory system known from animals [175]. For instance, intracellular pulsed Ca^2+^ signaling coordinates actin assembly and targeted exocytosis during hyphal tip growth [201] (see Sect. “Tip growth”), whereas extracellular Ca^2+^ pulses are involved in the cell-to-cell communication during germling fusion [109, 149].

### Tip growth

Tip growth is a highly polarized type of cell expansion resulting in a tube-like morphology with an apical dome in which growth processes take place. This growth habit is characteristic for the hyphae of filamentous fungi, but also found for some specialized cell types in plants and, in animals, exclusively present in neurons, where growth cones, large actin-supported extensions of the developing or regenerating neurite, search for their synaptic target. In fungi, hyphal tips harbor a characteristic structure, the Spitzenkörper (Fig. 4), which serves as an organizing center for hyphal tip growth [166, 167] (Fig. 4). In plants, tip growth is restricted to the protonema of mosses, rhizoids of the gametophytes of mosses and ferns, and root hairs and pollen tubes of seed plants [174] (Fig. 1B). The so-called growth cone serves an analogous role in neurite outgrowth during axon guidance. In each case, tip growth relies on cytoskeletal (re-)organization and orchestration of the machinery for polar secretion, indicating mechanistic parallels in tip growth mechanisms across eukaryotic kingdoms [148].

### Electrically excitable cells

Animals use, in addition to chemical cues, electrical signals for intra-organismal cell-to-cell communication. The major types of excitable cells comprise neurons (nerve cells) and muscle cells. Especially neurons are highly specialized for the long-distance transmission of signals via changes in the electrical potential along the axon—a long projection of neuronal cells that originates from the nerve cell body and interconnects neighboring neurons, often via dendrites (short branched protoplasmic projections of neurons). Within the myelinated axon, electrical impulses are propagated in the form of action potentials by saltatory conduction. Plants and fungi possess cells that are at least in part electrically excitable. Classical examples are stomatal guard cells [96] and the elongated parenchyma cells in the phloem and protoxylem of mimosa plants [191]. More recently, it was discovered that wounded leaves can communicate their damage status systemically by changes in the leaf surface potential [139].

Early investigations identified electrical currents generated by clustered ion channels and pumps in certain regions of fungal cells [70]. Transhyphal currents correlate with tip growth, albeit are not essential for apical cell extension per se [69, 132]. Electric currents across the plasma membrane are the basis for galvanotropic responses in conidial germlings and hyphae of several fungal species [22]. Recent studies even detected action potential-like spikes that propagate through mycelial networks [40]. Nevertheless, the molecular and cellular details of these phenomena remain largely unclear. The suspected roles for these spikes are in growth propagation, transport of nutrients and metabolites and—probably most significantly in terms of excitation—cellular communication. Electrical signals have been postulated to drive identity differentiation during germling fusion between isogenic cells of *N. crassa* [68] (see below).

### Tumor formation

Uncontrolled cell propagation in animals can result in the formation of often undifferentiated or dedifferentiated cell masses (tumors). Tumors are typically the result of a loss of cell cycle control, with often is conferred by the inappropriate activation of “proto-oncogenes” or the loss of function of “tumor suppressor genes” as a consequence of inherited, spontaneous and/or acquired mutational events in somatic cells [113], which besides spontaneous or induced mutations can also be conditioned by viral infections. Animal tumors can either reside locally or spread systemically through the body, leading to the formation of secondary tumors (metastases). The latter kind, also referred to as malignant tumors, is the hallmark of the group of diseases collectively termed as cancer (though some types of cancer, e.g. leukemias, are not associated with the formation of solid tumors). While fungi are not known to form tumors—probably due to the fact they can anyway grow indefinitely provided sufficient water and nutrients supply—plant tumors are a well-established phenomenon. They are frequently pathogen-induced, i.e. the consequence of bacterial, fungal, viral or insect infections, while spontaneous tumors are rare [46]. Prominent examples of tumors caused by pathogens are the crown galls induced by the soil bacterium *Agrobacterium tumefaciens* and the tumors induced by the corn smut fungus, *Ustilago maydis*. The rare occurrence of spontaneous plant tumors could be due to differences in cell cycle regulation as compared to animals, including a higher degree of genetic redundancy of key regulators found in some plants [47]. Additionally, the short life-span of herbaceous (non-woody plants) might prevent the accumulation of enough somatic mutations in crucial regulators of cell cycle or cell division. Rather, also for plant tumors not induced by pathogens, the changes driving the transition from normal growth to tumor formation are different as compared to animals and include disturbance of phytohormonal balance. In general, plant tumors are less lethal than their animal counterparts are. The physical constraints of plant cell walls (see Sect. “Extracellular matrix”) and the lack of a circulatory system (see Sect. “Long distance transport”) prevent the formation and spread of metastases within the plant body [47], which, in animals, give rise to the most deleterious consequences of tumors/cancers.

### Self and non-self recognition

The discrimination of self from non-self forms the basis of cellular and organismal immunity. Complex eukaryotes such as animals, plants and fungi have different strategies and deploy different receptor arsenals for the recognition and the combating of non-self. In vertebrates, two major lines of immunity are in place—innate immunity and adaptive immunity. Innate immune responses are the first line of defense, are primarily cell-autonomous and based on preformed anatomical barriers, phagocytes, secreted antimicrobial peptides and proteins, as well as cells that release inflammatory mediators [163]. Pathogen perception is largely mediated by germ line-encoded catalytically inactive Toll-like cell surface receptors and intracellular nucleotide-binding and oligomerization domain-like receptors (NLRs) [7]. Adaptive immunity relies on professional immune cells (B and T lymphocytes) and comprises the production of immunoglobulins (antibodies) by activated B cells and signaling as well as cytotoxic activities via T cells. Other than innate immunity, the system is highly adaptable since antibodies and T cell receptors are created by somatic genomic recombination and hypermutation events, providing an enormous diversity in recognition specificities [125]. Plants and fungi lack adaptive immunity and specialized immune cells. Instead, they fully rely on innate immunity.

Plants have a two-tiered innate immune system, with cell surface receptor-like kinases that perceive general molecular patterns (microbe-associated molecular patterns and danger-associated molecular patterns) and polymorphic cytoplasmic NLRs that directly or indirectly recognize pathogen-specific molecules (e.g. effector proteins) [97]. Similar to animal innate immunity, plant immunity is largely cell-autonomous and, thus, operates locally [45], but also has a systemic, phytohormone-mediated component [217]. Non-self recognition also plays a role in the context of self-incompatibility—a plant reproductive strategy that promotes cross-fertilization to maximize genetic diversity. Depending on the plant family, different male and female specificity determinants (encoded by the polymorphic *S* locus) interact upon the pollen-pistil contact during fertilization, subsequently triggering the self/non-self discrimination process [58].

Fungal immunity is even simpler and, as far as is known, mostly relies on cytosolic NLRs that may use similar mechanisms as animals and plants to recognize and respond to heterospecific non-self [209]. The general differentiation between self and non-self has essential roles at various stages of fungal development, most notably in sexual reproduction, genome defense and cell fusion. To recognize and home towards the opposite mating type partner, filamentous fungi use bipolar to tetrapolar sex pheromones and cognate pheromone receptor systems [107]. Vegetative non-self recognition is controlled via so called heterokaryon (*het*) genes and their incompatibility triggers PCD (see above) [66]. Additionally, non-self recognition can be triggered through the perception of foreign macromolecules, such as induced cell wall degradation products [73].

The most intriguing non-self recognition system in filamentous fungi, nevertheless, is the oscillatory recruitment of signaling proteins during self-fusion of isogenic conidial germlings of *N. crassa* [56]. Due to the repetitive exchange of signaling molecules between the two interacting cells, this phenomenon has also been termed the ‘ping-pong’ mode of homing [55, 160]. So far, over 60 proteins, several of them fungal-specific, have been identified to partake in non-self recognition and self-fusion [38, 223].

## Concluding remarks

Cell biology in current research and teaching shows an animal- or even mammalian-centric bias. This is on the one hand understandable as cell biology is one of the basics for comprehending human pathologies. On the other hand, this preference obscures the view of the huge diversity and full beauty of cell biology. Here, we have provided a comparative description of the cell biology of multicellular eukaryotic organisms emphasizing commonalities and differences (summarized in Table 1). For space reasons, this comparison could only highlight some key cell biological aspects but neglects the variation within each of the three kingdoms (in particular—but not only—in its early-diverged lineages), the enormous diversity of single cell organisms/protists, and execptions found in some organisms or specialized cell types. We nevertheless hope providing a useful starting point for researchers and students appreciating the diversity in cell biology also in the light of the current and future global challenges like maintaining biodiversity, dealing with climate crisis or enabling food security.

A marked difference between the eukaryotic lineages considered in this comparative review is their nutritional mode. While animals are heterotrophic, i.e. they are strictly reliant on obtaining their food and energy from other organisms, plants are largely (photo-)autotrophic, i.e., they produce organic materials by photosynthesis. By contrast, fungi are often saprophytic, i.e. they thrive on dead organic matter, but they also engage in parasitic or symbiotic lifestyles, with animals, plants and within their own kingdom alike. These different nutritional habits determine for example the composition of the respective extracellular matrices, as discussed above (see Sect. “Extracellular matrix”). Additional features of the considered eukaryotic lineages such as the occurrence of contractile cells (see Sect. “Contractile Cells”) may likewise relate to the mode of nutrition.

Current technical developments such as in single cell transcriptome sequencing, super resolution microscopy, cryoelectron microscopy and electron cryotomography allow for unprecedented insights into cellular organization and mechanisms and will predictably disclose more variances between the three kingdoms. We, thus, anticipate a wealth of further exciting insights into the diversity of eukaryotic cells, their structures and functions in the near future.

## Data Availability

Not applicable.

## Abbreviations

ECM: Extracellular matrix
GPCR: G-protein coupled receptor
MTOC: Microtubule-organizing center
NLR: Nucleotide-binding and oligomerization domain-like receptor
PCD: Programmed cell death
PtdIns: Phosphoinositides
RLK: Receptor-like kinase
